# An In Silico Identification of Common Putative Vaccine Candidates against *Treponema pallidum*: A Reverse Vaccinology and Subtractive Genomics Based Approach

**DOI:** 10.3390/ijms18020402

**Published:** 2017-02-14

**Authors:** Arun Kumar Jaiswal, Sandeep Tiwari, Syed Babar Jamal, Debmalya Barh, Vasco Azevedo, Siomar C. Soares

**Affiliations:** 1 Institute of Biological Sciences, Federal University of Minas Gerais, Belo Horizonte 31270-901, MG, Brazil; arunjaiswal1411@gmail.com (A.K.J.); sandip_sbtbi@yahoo.com (S.T.); syedbabar.jamal@gmail.com (S.B.J.); dr.barh@gmail.com (D.B.); vascoariston@gmail.com (V.A.); 2 Department of Immunology, Microbiology and Parasitology, Institute of Biological Sciences and Natural Sciences, Federal University of Triângulo Mineiro (UFTM), Uberaba 38025-180, MG, Brazil; 3 Centre for Genomics and Applied Gene Technology, Institute of Integrative Omics and Applied Biotechnology (IIOAB), Nonakuri, Purba Medinipur, West Bengal 721137, India

**Keywords:** sexually transmitted infections (STIs), drug target, vaccine target

## Abstract

Sexually transmitted infections (STIs) are caused by a wide variety of bacteria, viruses, and parasites that are transmitted from one person to another primarily by vaginal, anal, or oral sexual contact. Syphilis is a serious disease caused by a sexually transmitted infection. Syphilis is caused by the bacterium *Treponema pallidum* subspecies *pallidum*. *Treponema pallidum* (*T. pallidum*) is a motile, gram-negative spirochete, which can be transmitted both sexually and from mother to child, and can invade virtually any organ or structure in the human body. The current worldwide prevalence of syphilis emphasizes the need for continued preventive measures and strategies. Unfortunately, effective measures are limited. In this study, we focus on the identification of vaccine targets and putative drugs against syphilis disease using reverse vaccinology and subtractive genomics. We compared 13 strains of *T. pallidum* using *T. pallidum* Nichols as the reference genome. Using an in silicoapproach, four pathogenic islands were detected in the genome of *T. pallidum* Nichols. We identified 15 putative antigenic proteins and sixdrug targets through reverse vaccinology and subtractive genomics, respectively, which can be used as candidate therapeutic targets in the future.

## 1. Introduction

Sexually transmitted infections (STIs) are triggered by a number of bacteria, viruses, and parasites that are transferred mainly by vaginal, anal, or oral sexual contact between people. Different STIs can be existent or transmitted instantaneously, and such infections can trigger other STIs [1]. The World Health Organization (WHO) has reported more than 30 different bacteria, viruses, and parasites that are responsible for disease transmission through sexual contact.

Syphilis is among the most severe sexually transmitted infections (STIs) caused by the *Treponema pallidum* subspecies *pallidum*, a motile, gram-negative spirochete bacterium [2]. The annual estimated frequency of infectious syphilis is 36 million cases and over 11 million new infections; thus, it is an important public health burden globally [3]. Furthermore, the number of cases increased 10-fold in the last 15 years, with 4317 newly reported infections in 2014. This number is the highest it has been in the last 40 years and was mainly observed among men who have sex with men (MSM) [2].

If not properly treated, syphilis can cause long-term problems. It is important to screen women for syphilis during pregnancy to provide rapid treatment and to avoid congenital infections. Syphilis is a globally reemerging infection, as recently observed in the United States and Italy. Asian, African, and Latin American countries have high syphilis occurrences and are motivated to control prenatal care [4,5]. According to the Ministry of Health, in Brazil, 50,000 pregnant women are diagnosed with syphilis annually. The prevalence ranges from 1.1% to 11.5%, depending on maternal schooling and prenatal care. As a result, almost 12,000 infants are born with congenital syphilis each year [4]. In Brazil, the regulation of syphilis is one of the goals of the Pact for Health project initiated by the World Health Organization (WHO) for the elimination of congenital syphilis [4].

Despite sevendecades of penicillin use for the treatment of syphilis infections, *T. pallidum* exhibits complete sensitivity to this antibiotic. An increase in treatment complexity has led to the use of azithromycin as an oral antibiotic. However, over the last few decades, resistance against macrolides has been reported in many countries and at present, macrolides are not recommended for the cure or prophylaxis of syphilis [6]. The recent global prevalence of syphilis elicits a need for sustained preventive measures and strategies. Unfortunately, effective measures are inadequate. Relevant application of chemicals, antibiotics, lotions, creams, and thorough washing with soap and water after sexual contact are ineffective. The development of an effective vaccination appears to be the only alternative for the control of syphilis in the future. In spite of intense research for developing proper syphilis treatments, restricted progress has been noticed [7]. There are recent cases of emergence reported in several countries including Norway [8], China [9], the United States, Western Europe [10], and Martinique [11]. Although in today’s drug discovery process, high-throughput techniques and synthetic chemistry accelerate the process dramatically, it still takes 10–15 years to introduce a new drug to the market and therefore, a large investment is required [12].

The first step in the drug and vaccine discovery process is target identification. With the advent of new sequencing technologies and the deluge of genomic data, scientists are able to use computational methods to rapidly identify new targets, which are more time and cost effective than old approaches. Computational methods (i.e., subtractive genomics) are broadly used in this process. Recently, working with bacterial pathogens using an in silico approach, a large number of targets have been identified that are either resistant to drugs or for which no appropriate vaccine is available [13]. Reverse vaccinology is a conventional and popular approach in the post-genomic era for the prompt identification of novel vaccine targets [14,15]. Approaches, such as comparative and subtractive genomics and differential genome analyses [16], are being widely utilized for target identification in several human pathogens, including *Mycobacterium tuberculosis* [17], *Helicobacter pylori* [18], *Burkholderia pseudomallei* [19], *Pseudomonas aeruginosa* [20], *Salmonella typhi* [21], and *Neisseria gonorrhoeae* [22]. Generally, the principle behind these approaches is the identification of gene/protein targets that are essential for the survival of the pathogen but are not homologous to genes/proteins of the host [23]. Nevertheless, the identified targets may have a certain degree of homology with the host protein and are essential for the survival of the pathogen; hence, they can also be selected for structure-based selective inhibitor development as an additional molecular target. The differences in the active sites or other pockets with suitable druggability of the pathogenic protein could play an important role when compared to the host protein [24,25]. In this study, we mainly focus on the *in silico* identification of putative vaccine and drug targets against syphilis disease using reverse vaccinology and subtractive genomics. The goal was to identify plant-derived new lead antimicrobial compounds, and the proposed drug molecules show favorable interactions, lowered energy values, and high complementarity with the predicted targets.

## 2. Result and Discussion

**Figure 1:**
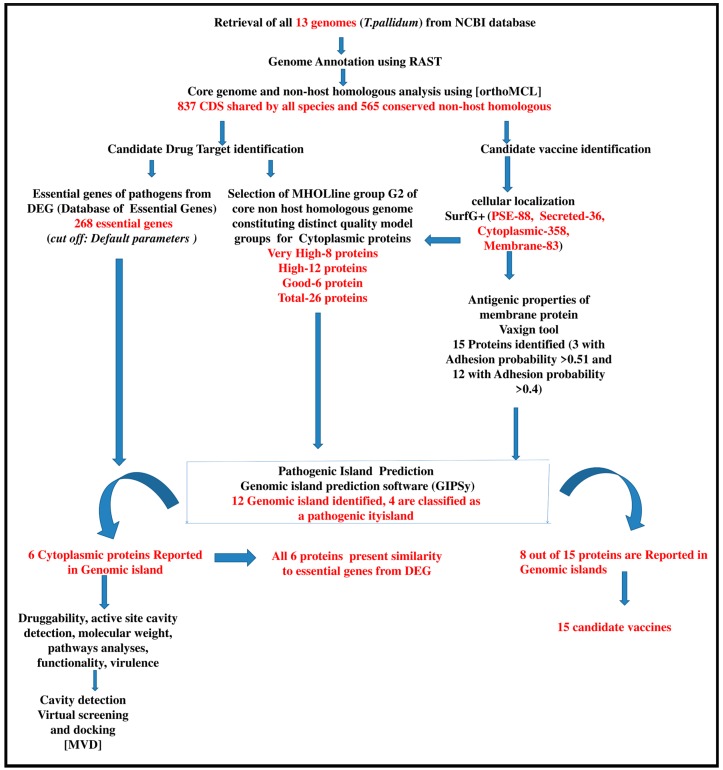
Complete workflow with the number of genes selected in each step and methodologies used. The sentences in black describe the analyses made and the software used in each step. The sentences in red represent the number of proteins selected in each step. CDS = coding DNA sequence; MVD = Molegro Virtual Docker.

The total number of proteins described in each of the following sections and all the methodologies used in our work are described on the workflow in Figure 1.

### 2.1. Identification of Intra-Species Conserved Non-Host Homologous Proteinsand Pathogenicity Islands

**Figure 2:**
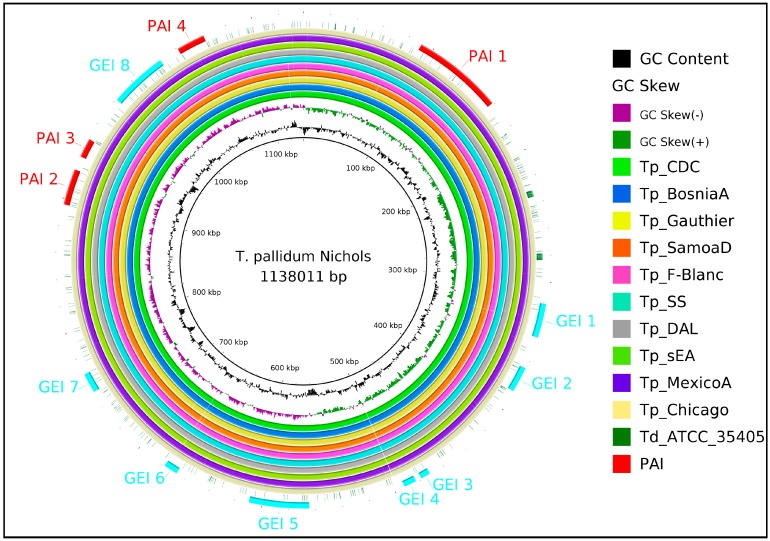
Genomic islands (GIs) of *T. pallidum* Nichols strains as predicted by the genomic island prediction software (GIPSy) using *Treponema denticola* as a closely related non-pathogenic organism. The outermost circle highlighted in red shows the four pathogenicity islands from 10 GIs. Guanine-Cytosine (GC) content is shown in black.

We compared 13 *Treponema pallidum* strains (Table 1) using *Treponema pallidum* Nichols as the reference using the orthoMCL software [26]. Coding DNA sequences (CDSs) shared by all species were considered a part of the core genome. Considering the human genome as the host genome, a set of 565 conserved non-host homologous proteins were identified. The prediction of genomic islands (GIs) was subsequently performed. GIs are gene clusters, usually >8 kb in size, likely acquired via horizontal gene transfers (HGT), and often playing a role in the environmental or host adaptation of bacteria. GIs significantly influence bacterial evolution and provide further insight in differentiating bacterial species and strains. For *T. pallidum* Nichols strains, 10 putative GIs were identified through the Genomic Island Prediction Software (GIPSy) [27], using *Treponema denticola* as a closely related, non-pathogenic organism. Of the 10 GIs, four are classified as pathogenicity islands (PAIs), i.e., they present high concentrations of virulence factors and are absent in the aforementioned closely related non-pathogenic organism (Figure 2).

### 2.2. Assessment of Essential Genes

Essentiality analysis identifies significant genes required for pathogen survival such as adhesion, entry into the host, infection, and persistence in the host [13]. The conserved 565 non-hosts homologous proteins were subjected to the Database of Essential Genes (DEG) for the identification of essential proteins, through which a final set of 268 proteins was obtained (Table S1). Essential proteins are necessary for the survival of pathogen within the host. When these essential proteins are declared to be virulent, they can be of vital significance to unveil novel therapeutic targets. There is a probability of essential proteins to be conserved among various populations and species because of their vital roles in various pathways for pathogen survival [13,28]. Virulence is the characteristic of a pathogen responsible for causing severe human diseases. In the present study, these properties have been given high priority to identify potential vaccine candidates computationally. Although only 268 proteins were identified as essential by DEG, we considered all 565 proteins for our analyses.

### 2.3. Prediction of Candidate Vaccine Target for T. pallidum

The subcellular localization of conserved non-hosts homologous proteins of *T. pallidum* strains were predicted with the SurfG+ software [29]. We classified 207 gene products as putative surface-exposed (PSE) proteins, secreted proteins, or membrane proteins (Table 2). The proteins predicted by SurfG+ were further analyzed with the software Vaxign [30] for antigenic properties with adhesion probabilities greater than 0.51, resulting in the detection of three proteins in the *T. pallidum* strains Nichols (Table 3). We found that out of these three proteins, Tp_Nichols141 and Tp_Nichols797 were hypothetical proteins. Tp_Nichols141 belongs to the pathogenicity island 1 (Figure 2). When the adhesion probability threshold was >0.4, we also identified 12 more proteins that can also be considered potential vaccine candidates against *T. pallidum.*

Previous studies have shown the importance of targeting proteins involved in the capability of *T. pallidum* to invade host tissues and to evade the functional immune response, contributing to its persistence during the “latency” stage. Most of the described gene targets code for proteins responsible for the attachment to extracellular matrix bridges (Tp0136, TP0155, Tp0483, and Tp0751), such as the low density integral Outer Membrane Proteins (OMPs) [6]. Briefly, in our predictions of good vaccine targets, we have identified Tp_Nichols350 and TpNichols852 with similarities to two previously described OMPs (TP0453 and Tp_0326), along with two additional OMP domain containing proteins: Tp_Nichols797 and Tp_Nichols141. Interestingly, both Tp_Nichols797 and Tp_Nichols141 presented adhesion probabilities higher than 0.5 and should be given priority in in vitro assays.

### 2.4. High Throughput Structural Modeling

The main focus of this study was to find candidate vaccine targets. However, according to Caroline et al., 2014 [6], the difficulty in curing syphilis is due to the vilification of many antibiotics for treatment or prophylaxis. Our contributionsincludetheprediction of some novel drug targets against *Treponema pallidum.* For this, the identified 565 conserved non-host homologous *Treponema pallidum* proteins were submitted to MHOLline [31] an online web tool, to predict the modelome. MHOLline utilizes multi-fasta files of amino acids as an input data and then uses HMMTOP, BLAST, BATS, MODELLER, and PROCHECK programs for the detailed analyses. The program HMMTOP detects transmembrane regions. The BLAST algorithm is used to identify the template structure by performing a random search against the Protein Data Bank. BATS (Blast Automatic Targeting for Structures) carries out the refinement in the template search and it is a key step for the model construction. BATS refinement identifies sequences that make the modeling possible by selecting a template from a BLAST output file using their BATS scores, expectation values, identity, and sequence similarity as criteria, as well as considering the number of gaps and the alignment coverage. BATS selects the best template for 3D model generation and performs automated alignment using the MODELLER program. Furthermore, it gathers all the BLAST output files into four distinctive groups (i.e., G0, G1, G2, and G3) according to the following criteria: G0 = unaligned sequence; G1 = E-value > 10 × 10^−5^ or identity <15%; G2 = E-value ≤ 10 × 10^−5^ and identity ≥25% AND LVI ≤ 0.7; G3 = E-value ≤ 10 × 10^−5^ and identity ≤15% and <25% OR LVI (Length Variation Index) >0.7. Only the first three distinct quality G2 model groups were taken into consideration in this study; these were: 1—very high quality model sequences (identity ≥75%) (LVI ≤ 0.1), 2—high quality model sequences (identity ≥50%) and <75%) (LVI ≤ 0.1), and 3—good quality model sequences (identity ≥50%) (LVI > 0.1 and ≤0.3) [31]). Therefore, all the considered protein 3D models were constructed from sequences for which their template is available with identity ≥50%. We found 26 proteins (8 very high, 12 high, and 6 good) in the first 3 distinct quality G2 model groups.

The membrane and cell wall associated proteins are, theoretically, more exposed as targets than the cytoplasmic drug targets. However, membrane proteins are difficulty to purify and assay [32]. Cytoplasmic membrane proteins are also very important for the physiology of bacteria, as they are involved in many important metabolic functions. Therefore, the membrane, putative surface exposed, and secreted proteins are better applicable as targets for reverse vaccinology, whereas the pivotal role of cytoplasmic proteins in maintenance of cell viability makes them more favorable as drug targets [33]. Out of the 26 proteins, only cytoplasmic proteins that were present in any GIs were selected as candidate drug targets. Six proteins that were also present in the 268 proteins were identified as essential in the DEG analyses and were considered for the target prioritization and docking studies (Table 4).

The outer membrane may pose a barrier for drugs to gain access to cytoplasmic targets. However, small molecules are able to gain access to the periplasm through porins and reach the cytoplasm. In previous studies, it was shown that one of the pore forming OMPs, OmpF, has an exclusion limit of 600 Daltons, for example, which is used by ions, amino acids, and small sugars as a means to reach the periplasm [34]. The molecular weight of the compounds used here varies from ~275.1 g/mol (liriodenine) to~488.7 g/mol (jacarandic acid) and they may also be able to use porins to gain access to the periplasm. Alternatively, the use of nanoparticles as delivery systems or a combined treatment, such as with polymyxins and derivatives that increase the permeability of the outer membrane, may also help in overcoming the outer membrane barrier [35].

### 2.5. Analyses of Non-Host Homologous Targets and Molecular Docking

**Figure 3:**
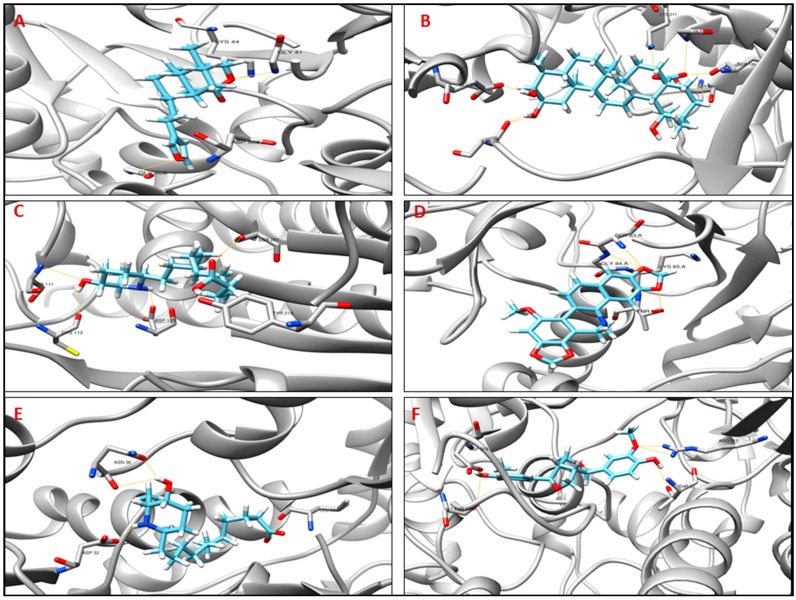
3D graphic representation of the docking analyses for the most druggable protein cavity of drug target. (**A**) Tp_Nichols130 (uvrB, Uvr ABC system protein B) with potamogetonin (CID 5742898); (**B**) Tp_Nichols593 (pfp, pyrophosphate—fructose 6-phosphate 1-phosphotransferase) with jacarandic acid (CID 73645); (**C**) Tp_Nichols609 (asnA, aspartate-ammonia ligase) with leptophyllin B (CID 10447482); (**D**) Tp_Nichols754 (recA, RecA protein) with dihydrochelirubine (CID 440589); (**E**) Tp_Nichols9904 (ndh, NADH dehydrogenase) with leptophyllin B (CID 10447482); (**F**) Tp_Nichols1011 (dxs 1-deoxy-d-xylulose-5-phosphate synthase) with pinoresinol (CID 234817).

In molecular docking, lower energy scores represent better protein-ligand bindings compared to higher energy values [37]. We considered the lower MolDock score and the interaction with the residues that were involved in the active site of the target for the prediction of therapeutic candidates. For each target protein (uvrB, pfp, asnA, recA, ndh, and dxs), a library of 28 natural compounds were docked to examine each molecule one-by-one for the selection of the final set of promising molecules that showed favorable interactions with the active site residues of targets. The biological importance for each target is described here (Table 4) along with an analysis of the predicted protein-ligand interaction(s). The name of the molecules, MolDock scores for the selected ligands, and the number of predicted hydrogen bonds with the active residues involved in these interactions are shown below for each target protein (Table 5). The predicted configurations of one of the best-docked molecules are also shown for each pathogen target in Figure 3A–F.

Based on a structural comparison with a crystallographic structure of the uvrB template (2d7d, uvrB from *Bacillus subtilis*), the active site residues involved in H-bond interactions with the crystallographic ligand adenosine-5′-diphosphate are Phe10, Gln11, Gln16, Gly41, Gly43, and Arg541. One of these residues, Gly41, was predicted to make hydrogen bonds to the ligand potamogetonin (CID 5742898) with a MolDock score of −97.81. Similarly, for the target pfp template (2F48, *Borrelia burgdorferi*), the active site residues involving in H-bond interactions are Lys211, Pro210, Asp214, Gly90, Tyr434, Arg154, Met259, Arg261, and Glu320. The residue Lys211 interacts with jacarandic acid (CID 73645) and pinoresinol (CID 234817) with MolDock scores of −62.15 and −112.67, respectively. The compound leptophyllin B (CID 10447482) interacts with the identified active site residues Ser111, Cys113, Asp115, Tyr218, and Ser251of asnA (PDB ID: 12AS from *Escherichia coli*) and Leu298, Asp32, and Asn36 of ndh (PDB Template ID: 2BC0 from *Streptococcus pyogenes*).

Interestingly, the drug molecule pinoresinol (CID234817) was predicted to show good results against four of our targets uvrB, pfp, asnA, and dxs. Pinoresinol is a lignan, biphenolic compound found in *Araucaria araucana* and *Sambucus williamsii.* It possesses bactericidal and fungicidal activities and therapeutic potential as an antifungal agent for the treatment of fungal infectious diseases in humans [38,39]. Thus, the identification of pinoresinol in our in silico study strengthens our protocol and can be potentially used as a new drug for the treatment of syphilis.

## 3. Materials and Methods

### 3.1. Selection of Data

The genome sequences of all 13 strains of *T. pallidum* were retrieved from the NCBI (National Center for Biotechnology Information) server [40]. For homogeneity in the functional annotation, all genomes were annotated using the RAST server (RapidAnnotationsusing SubsystemsTechnology) [41]. Furthermore, these annotated genome sequences were used for analysis.

### 3.2. Identification of Intra-Species Conserved Non-Host Homologous Proteins

In comparative genomics, the orthologous genes are clustered to obtain a framework to integrate information from multiple genomes, highlighting the conservation and divergence of gene families and biological processes. For pathogens, clustering orthologs can facilitate drug and/or vaccine targets identification. We compared 13 strains of *Treponema pallidum* using *Treponema pallidum* Nichols as the reference genome, using orthoMCL software [26] with an E-value of 1 × 10^−50^. CDSs shared by all strains were considered a part of the core genome. The possible candidates for drugs and/or vaccines should be non-homologues to human proteins; thus, autoimmunity is avoided, and an accurate immune response is elicited against the targeted pathogen. Accordingly, these core genes were subjected to orthoMCL software (E-value = 1 × 10^−50^) against the human genome for the identification of non-host homolog targets.

### 3.3. Identification of Pathogenicity Islands

Knowledge about pathogenicity islands, the virulence factors they encode, their mobility, and their structure is not only helpful in understanding the bacterial evolution and their interactions with eukaryotic host cells, but may also facilitate in providing delivery systems for vaccination and tools for the development of new approaches for treating bacterial infections [28]. The identification of pathogenicity islands in the genome of *T. pallidum* Nichols was performed with GIPSy (Genomic Island Prediction Software) [27] through the detection of regions presenting: deviations in genomic signature (i.e., anomalous G+C and/or codon usage deviation); presence of transposase, virulence or flanking tRNA genes; and absence in the non-pathogenic organism *Treponema denticola.*

### 3.4. Assessment of Essential Genes

A subtractive genomics approach was followed to identify conserved targets that were essential to the bacteria [13]. The set of core conserved proteins of *T. pallidum* Nichols was subjected to the Database of Essential Genes (DEG) [42] for homology analyses. The DEG contains experimentally validated data from bacteria, archaea, and eukaryotes that are comprised of currently reported essential genomic elements including protein-coding genes that are indispensable to support cellular life. The cut-off values used for BLASTp were: E-value = 0.0001, bit score =100, and identity = 25% [15,18,30].

### 3.5. Reverse Vaccinology Approach for Prediction of Putative T. pallidum Vaccine Targets

For potential vaccine targets, subcellular localization and the secretion of pathogenic proteins are important factors for consideration, where secreted and membrane proteins are the first to be in contact with the host, eliciting an immune response. Therefore, the prediction of the exoproteome or secretome, composed of the proteins localized in the extracellular matrix or outer membrane of the organism, is highly valuable for reverse vaccinology strategies. In combination with subtractive proteomics, reverse vaccinology can provide a more reliable output compared to screening of the whole data set without considering prioritizing parameters [13]. The non-host homologous conserved proteome of *T. pallidum* Nichols was screened using SurfG+ software [29] to identify secreted proteins, membrane proteins, and putative surface exposed proteins. We searched for cleavage sites and transmembrane helices in all 15 proteins using SignalP [43] and TMHMM (Transmembrane Helix prediction server, based on a hidden Markov model) [44], respectively, and we also predicted the presence of functional domains for all the 15 proteins with InterProScan, which uses several databases for domain prediction [45]. The dataset was screened by Vaxign [30] by searching for proteins with the following features: major histocompatibility complex (MHC I) and (MHC II) binding properties, an adhesion probability greater than 0.51, and no similarity to host proteins.

### 3.6. High Throughput Structural Modeling

MHOLline [31] was used to predict the modelome (complete set of protein 3D models for the whole conserved core non-host homologous proteome). MHOLline utilizes multi-fasta files of amino acids as input data and then uses HMMTOP, BLAST, BATS, MODELLER, and PROCHECK programs for the detailed analyses. The program HMMTOP detects transmembrane regions [46]. The BLAST algorithm is used to identify template structure by performing random searches against the Protein Data Bank [47]. BATS (Blast Automatic Targeting for Structures) performs the refinement in the template search; its use represents a key step for the model construction. BATS refinement identifies sequences that make the modeling possible by selecting templates from the BLAST output file using their BATS scores, expectation values, identity, and sequence similarity as criteria as well as considering the number of gaps and the alignment coverage. BATS selects the best template for 3D model generation and performs automated alignment used by the MODELLER program. The adopted methodology was revised accordingly from the original work by Hassan et al. [46].

### 3.7. Ligand Libraries and Docking Analyses

The ligand libraries of 28 natural compounds presented by Tiwari et al., 2014 [48] were used for the docking analysis. The 3D structures of all target proteins were carefully examined for structural errors (wrong bonds, missing atoms, and protonation states) in the MVD (Molegro Virtual Docker) [37]. The active side residues of the target proteins were identified by comparing its 3D structure to the respective templates. Furthermore, taking identified cavities from a template used in a grid for molecular docking. The program includes three search algorithms for molecular docking analyses, namely MolDock Optimizer [37], MolDock Simplex Evolution (SE), and Iterated Simplex (IS). We employed the MolDock Optimizer search algorithm, which is based on a differential evolutionary algorithm, using the default parameters, that are (a) population size = 50; (b) scaling factor = 0.5; and (c) crossover rate = 0.9. The 3D poses of docked molecules were analyzed in Chimera [49]. Molecular function (MF) and biological process (BP) for each target protein were determined using UniProt [41]. The biochemical pathway of these proteins were checked using KEGG (Kyoto Encyclopedia of Genes and Genomes) [50], SurfG+ software [29], and virulence using GIPSy [31]. The final list of targets was based on 12 criteria, as described earlier in [13,46].

## 4. Conclusions

Here, the genomic information was used with the aim of determining the conserved proteome of 13 strains of *Treponema pallidum* in a search for regions of genome plasticity. Moreover, we used reverse vaccinology and subtractive genomics to predict new antigenic/drug targets, which can be used in the development of new vaccines and drugs for *Treponema pallidum.* After a detailed in silico analysis between host and pathogen proteins, we suggest that the identified non-host homologous proteins could be considered for prophylaxis of syphilis due to further experimental validations.

## Figures and Tables

**Table 1:** Genomic features of all *T. pallidum* (Tp) strains.

Strain	Size (Mb)	GC%	Gene	Protein
Tp_Nichols	1.13	52.80	1044	970
Tp_Sea81-4	1.13	52.80	1032	931
Tp_SS14	1.13	52.80	1042	971
Tp_Chicago	1.13	52.80	1030	969
Tp_SamoaD	1.13	52.80	1027	971
Tp_CDC2	1.13	52.80	1030	973
Tp_Gautheir	1.13	52.80	1029	971
Tp_DAL1	1.13	52.80	1030	969
Tp_MexicoA	1.14	52.80	1029	968
Tp_Fribourg-Blanc	1.14	52.80	1030	970
Tp_SS14 (14.8.2015)	1.13	52.80	1029	970
Tp_BosniaA	1.13	52.80	1027	970
Tp_pallidum	1.13	52.70	1033	964

**Table 2:** Subcellular location of Treponema pallidum (Tp) strain proteins.

Localization	Number of Proteins
Cytoplasmic Protein	358
Membrane Protein	83
PSE ^a^	88
Secreted Protein	36

^a^ Putative Surface Exposed.

**Table 3:** Putative antigenic proteins of *Treponema pallidum* (Tp) identified using Vaxign.

Tp_Nichols	Protein ID	Gene Name	Subcellular Localization	SignalP Result (Cleavage Site)	TMHMM Result	InterProScan (Domain)	Gene Product	Adhesion Probability
Tp_Nichols797	WP_010882178.1	-	SEC	Yes (between 25 and 26)	TMH = 0	Outer membrane protein/outer membrane enzyme PagP, beta-barrel—IPR011250 (65–219)	Hypothetical protein	0.552
Tp_Nichols141	WP_014342713.1	-	PSE	No	TMH = 1	Outer membrane protein/outer membrane enzyme PagP, beta-barrel—IPR011250 (100–225)	Hypothetical protein	0.525
Tp_Nichols466	WP_010881878.1	*ntpK*	MEM	No	TMH = 4	V-ATPase proteolipid subunit C-like domain—IPR002379 (76–138)	Two-sector ATPase, V(0) subunit K	0.590
Tp_Nichols930	WP_010882306.1	*slyD*	PSE	No	TMH = 1	Peptidyl-prolyl cis-trans isomerase, FKBP-type, N-terminal—IPR000774 (66–143)	FKBP-type peptidyl-prolyl cis-trans isomerase SlyD	0.488
Tp_Nichols471	WP_010881883.1	*nlpE*	SEC	Yes (between 23 and 24)	TMH = 0	No	Copper resistance lipoprotein NlpE	0.475
Tp_Nichols650	WP_010882040.1	-	PSE	No	TMH = 2	Domain of unknown function DUF2147—IPR019223 (71–193)	Hypothetical Protein	0.474
Tp_Nichols1046	WP_010882416.1	*ftr1*	MEM	No	TMH = 6	No	Conserved hypothetical integral membrane protein	0.44
Tp_Nichols52	WP_010881498.1	*TPANIC_0600*	PSE	No	TMH = 1	Duplicated hybrid motif—Ipr011055 (196–355)	Zinc metalloprotease	0.428
Tp_Nichols610	WP_010882004.1	-	SEC	No	TMH = 1	Zinc finger, CHCC-type—IPR019401 (8–34)	Hypothetical Protein	0.425
Tp_Nichols323	WP_010881746.1	-	SEC	No	TMH = 1	Sporulation-related domain—IPR007730 (172–252)	Hypothetical Protein	0.41
Tp_Nichols852	WP_010882234.1	*TP_0453*	SEC	Yes (between 23 and 24)	TMH = 0	No	Outer membrane protein TP0453	0.408
Tp_Nichols350	WP_014342788.1	*tp92*	SEC	Yes (between 37 and 38)	TMH = 1	Bacterial surface antigen (D15)—IPR000184 (478–849)	Putative outer membrane protein assembly factor TP_0326	0.405
Tp_Nichols98	WP_010881537.1	-	PSE	No	TMH = 0	No	Hypothetical Protein	0.401
Tp_Nichols347	WP_010881771.1	*TP_0323*	MEM	No		No	Ribose/galactose ABC transporter, permease protein (RbsC-2)	0.401
Tp_Nichols362	WP_010881783.1	*TPANIC_0335*	MEM	No	TMH = 2	No	Putative membrane protein	0.401

SEC = secreted; PSE = Putative surface exposed; MEM = Membrane; TMH = Transmembrane Helix, TMHMM = Transmembrane Helix prediction server, based on a hidden Markov model.

**Table 4:** Drug target prioritization parameters and functional annotation of the six non-homologous putative targets.

Locus Tag, Gene, and Protein ID	Official Full Name	Mol. Wt (KDa) ^a^	Functions ^b^	Cellular Component ^c^	Pathways ^d^	Virulence ^e^	DEG Analyses
Tp_Nichols130, uvrB, WP_010881565.1	UvrABC system protein B	76.19	MF: ATP (Adenosine triphosphate) binding, DNA binding, excinuclease ABC activity, helicase activity. BP: nucleotide-excision repair, SOS response.	Cytoplasm	Unknown	Yes	Essential gene
Tp_Nichols593, Pfp, WP_010881989.1	Pyrophosphate-fructose 6-phosphate 1-phosphotransferase	62.43	--	Cytoplasm	Glycolysis	Yes	Essential gene
Tp_Nichols609, asnA, WP_010882003.1	Aspartate-ammonia ligase	36.86	MF: Aminoacyl-tRNA ligase activity, aspartate-ammonia ligase activity, ATP binding.BP: l-asparagine biosynthetic process, tRNA aminoacylation for protein translation.	Cytoplasm	l-asparaginebiosynthesis	Yes	Essential gene
Tp_Nichols754, recA, WP_010882137.1	Protein RecA	45.33	MF: ATP binding, damaged DNA binding, DNA-dependent ATPase activity, single stranded DNA binding.BP: DNA recombination, DNA repair, SOS response.	Cytoplasm	Unknown	Yes	Essential gene
Tp_Nichols990, Ndh, WP_010882364.1	NADH (Nicotinamide adenine dinucleotide) dehydrogenase	48.64	MF: flavin adenine dinucleotide binding, NADH dehydrogenase activity.BP: cell redox homeostasis.	Cytoplasmic	Unknown	Yes	Essential gene
Tp_Nichols1011, Dxs, WP_010882382.1	1-deoxy-d-xylulose-5-phosphate synthase	129.82	MF: 1-deoxy-d-xylulose-5-phosphate synthase activity, magnesium ion binding, thiamine pyrophosphate binding.BP: 1-deoxy-d-xylulose-5-phosphate biosynthetic process, terpenoid biosynthesis process, Thiamine biosynthesis process.	Cytoplasmic	1-deoxy-d-xylulose 5-phosphate biosynthesis	Yes	Essential gene

^a^ Molecular weight was determined using the ProtParam tool [36]; ^b^ Molecular function (MF) and biological process (BP) for each target protein was determined using UniProt; ^c^ Cellular localization of pathogen targets was performed using SurfG+; ^d^ KEGG (Kyoto Encyclopedia of Genes and Genomes) was used to find the role of these targets in different cellular pathways; ^e^ PAIDB (PAthogenisity Island DataBase) and GIPSy were used to check if the putative targets are involved in pathogen virulence. DEG = Database of Essential Genes; MF = Molecular function; BP = Biological process.

**Table 5:** The MolDock scores of natural compounds and predicted hydrogen bonds for the selected best-ranked molecules against each drug target.

Compounds Name	MolDock Score	Number of H-Bond	Residues Interacting
Tp_Nichols130 (UvrB, Uvr ABC System Protein B)			
Diospyrin (CID 308140) MW: ~374.3 g/mol	−119.83	4	Gly506, Asp508
Pinoresinol (CID 234817) MW: ~358.4 g/mol	−114.82	2	His64, Asp508
Potamogetonin (CID 5742898) MW: ~314.4 g/mol	−97.81	4	Gly41, Lys44, Gly506, Asp508
Tp_Nichols593 (pfp, Pyrophosphate-fructose 6-phosphate 1-phosphotransferase)			
Pinoresinol (CID 234817) MW: ~358.4 g/mol	−112.67	5	Ser88, Lys211, Gly260, Glu320
Jacarandic acid (CID 73645) MW: ~488.7 g/mol	−62.15	7	Ser88, Ser186, Gly183, Lys211, Glu320, Ser396
Texalin (CID 473253) MW: ~266.3 g/mol	−91.57	4	Gly90, Thr212, Ser186, Ile213
Tp_Nichols609 (asnA, Aspartate-ammonia ligase)			
Leptophyllin B (CID 10447482) MW: ~299.4 g/mol	−141.21	5	Ser111, Cys113, Asp115, Tyr218, Ser251
Pinoresinol (CID 234817) MW: ~358.4 g/mol	−132.814	5	Ser49, Lys77, Ser251, Arg255
Liriodenine (CID 10144) MW: ~275.1 g/mol	−95.65	2	Lys77, Arg255
Tp_Nichols754 (recA, Protein RecA)			
Dihydrochelirubine (CID 440589) MW: ~363.4 g/mol	−138.94	4	Gly84, Lys85, Ser83, Thr86
Piperine (CID 638024) MW: ~285.3 g/mol	−17.14	5	Ser83, Gly84, Lys84, Gln207, Gly279
Rhein (CID 10168) MW: ~284.2 g/mol	−96.11	7	Ser83, Gly84, Thr86, Tyr116, Asn254, Gly279
Tp_Nichols990 (ndh, NADH dehydrogenase)			
Leptophyllin B (CID 10447482) MW: ~299.4 g/mol	−122.62	4	Leu298, Asp32, Asn36
Dicentrinone (CID 177744) MW: ~335.3 g/mol	−111.09	4	Arg33, Ala11
Isosakuranetin (CID 160481) MW: ~286.3 g/mol	−109.35	3	Arg33, Ala11, Cyc42
Tp_Nichols1011 (dxs, 1-deoxy-d-xylulose-5-phosphate synthase)			
Pinoresinol (CID 234817) MW: ~358.4 g/mol	−146.18	5	Asp978, Thr1006, Thr32, Arg115
Piperine (CID 638024) MW: ~285.3 g/mol	−131.40	3	Thr32, Arg115, Trp980
Berberine (CID 2353) MW: ~336.4 g/mol	−115.94	3	Thr32, Gly979, Asn1011

MW = molecular weight; CID = PubChem Compound Identifier.

## Supplementary Materials

Supplementary materials can be found at www.mdpi.com/1422-0067/18/2/402/s1.
